# Archaeal NSUN6 catalyzes m^5^C72 modification on a wide-range of specific tRNAs

**DOI:** 10.1093/nar/gky1236

**Published:** 2018-12-12

**Authors:** Jing Li, Hao Li, Tao Long, Han Dong, En-Duo Wang, Ru-Juan Liu

**Affiliations:** 1 State Key Laboratory of Molecular Biology, CAS Center for Excellence in Molecular Cell Science, Shanghai Institute of Biochemistry and Cell Biology, Chinese Academy of Sciences; University of Chinese Academy of Sciences, 320 Yueyang Road, Shanghai 200031, P.R. China; 2 School of Life Science and Technology, ShanghaiTech University, 100 Haike Road, Shanghai 201210, P.R. China

## Abstract

Human NOL1/NOP2/Sun RNA methyltransferase family member 6 (hNSun6) generates 5-methylcytosine (m^5^C) at C72 of four specific tRNAs, and its homologs are present only in higher eukaryotes and hyperthermophilic archaea. Archaeal NSun6 homologs possess conserved catalytic residues, but have distinct differences in their RNA recognition motifs from eukaryotic NSun6s. Until now, the biochemical properties and functions of archaeal NSun6 homologs were unknown. In archaeon *Pyrococcus horikoshii* OT3, the gene encoding the NSun6 homolog is *PH1991*. We demonstrated that the PH1991 protein could catalyze m^5^C72 formation on some specific *Ph*tRNAs *in vitro* and was thus named as *Ph*NSun6. Remarkably, *Ph*NSun6 has a much wider range of tRNA substrates than hNSun6, which was attributed to its tRNA substrate specificity. The mechanism was further elucidated using biochemical and crystallographic experiments. Structurally, the binding pocket for nucleotide 73 in *Ph*NSun6 is specific to accommodate U73 or G73-containing *Ph*tRNAs. Furthermore, *Ph*NSun6 lacks the eukaryotic NSun6-specific Lys-rich loop, resulting in the non-recognition of D-stem region by *Ph*NSun6. Functionally, the m^5^C72 modification could slightly promote the thermal stability of *Ph*tRNAs, but did not affect the amino acid accepting activity of *Ph*tRNAs.

## INTRODUCTION

As linker molecules between ribosomes and growing polypeptide chains, transfer RNAs (tRNAs) play a central role in protein synthesis. Recently, further functions of tRNAs have been identified in addition to translation (1–3). Multiple modifications are a characteristic property of tRNAs, and these modifications are crucial for tRNA stability, decoding accuracy, and cellular functions (4–8).

In contrast to tRNA modifications in prokaryotes and eukaryotes, study of archaeal tRNA modifications lags behind in aspects of genetic characterization and *in vivo* functions; however, archaeal tRNA modifications are indispensable (9,10). Archaeosine is exclusively found in most of known archaeal tRNAs at position G15 in the D-loop, ensuring the tRNA tertiary structure by tightening the tertiary base pair, G15:C48 (11,12). An agmatidine modification at the first anticodon position of C34 in archaeal tRNA^Ile^(CAU) is essential for precise decoding (13,14). Archaea-specific isowyosine and 7-methylwyosine, which are guanosine-37 derivatives, demonstrate the complexity in archaeal wyosine derivatives synthesis (15–20).

5-methylcytosine (m^5^C) is a ubiquitous modification in DNA and different cellular RNAs, which is formed by site-specific methyltransferases (MTases). The NSun family, which contains an S-adenosyl-methionine (SAM)-dependent NOL1/NOP2/Sun (NSun) domain, is the main RNA:m^5^C MTase and comprises seven family members in eukaryotes. Among them, NSun2, NSun3 and NSun6 are primarily responsible for m^5^C modifications on tRNAs (21–23). Genetic mutations and aberrant expression of several NSun family members are linked to human diseases and disorders, including intellectual disability (24,25), mitochondrial deficiency (26), Williams–Beuren syndrome (27), male infertility (28), and cancer development (29,30). In archaea, m^5^C modifications are widespread in cellular RNAs, especially in tRNAs. Positions C34, C38, C48, C49 and C50 of tRNAs are the known m^5^C modification sites in archaea (31,32).

m^5^C72 is a newly identified modification in the acceptor stem region of human cytoplasmic tRNA^Thr^ and tRNA^Cys^, established by human NSun6 (hNSun6) (23). Our previous study showed that well-folded and sequence-specific tRNA substrates are strictly selected by hNSun6 (33). The structure of hNSun6 in complex with tRNA revealed that the MTase domain works together with pseudouridine synthase and archaeosine transglycosylase (PUA) domain to recognize regions in the acceptor and D-stem of tRNAs (34). Homologs of hNSun6 are present only in higher eukaryotes and hyperthermophilic archaea. In contrast to the well-studied identified genes that encode eukaryotic NSun6s, the corresponding genes in archaea are unknown.

In the genome of *Pyrococcus horikoshii* OT3 (*P. horikoshii*), five genes encode putative RNA m^5^C MTases: *PH1537, PH1374, PH1078, PH1991*, and *PH0851*. Among them, PH1991 shares the highest sequence similarity (23.9% identity) with hNSun6, suggesting that PH1991 is the hNSun6 homolog. Interestingly, compared with hNSun6, the amino acid sequence of PH1991 is shorter and the predicted RNA recognition residues, especially the predicted PUA domain (residues 86–164) and RNA recognition motifs (RRMs, residues 34–79 and 173–185), are extremely divergent from eukaryotic NSun6s (Supplementary Figure S1). Moreover, PH1991 lacks the eukaryotic NSun6-specific conserved Lys-rich loop in the PUA domain, which is essential for D-stem recognition by eukaryotic NSun6s. Until now, the properties and functions of PH1991 were uncharacterized. Thus, the present study aimed to determine whether PH1991 retains the m^5^C MTase catalytic activity; what are the RNA substrates of PH1991, considering the differences in the predicted RNA binding residues; and what is the biological function of PH1991.

Moreover, NSun6 homologs are conserved in other hyperthermophilic archaea, such as *Pyrococcus kukulkanii* NCB100 (35), *Pyrococcus abyssi* GE5 (http://www.genoscope.cns.fr/Pab/), *Thermococcus litoralis* NS-C (36) and *Thermococcus nautili* 30-1 (37). These archaeal NSun6 homologs all share identity with PH1991 (Supplementary Figure S1). However, the biochemical properties and functions of PH1991 analogs are unknown.

In this study, we identified that PH1991 is a tRNA:m^5^C72 MTase NSun6 (*Ph*NSun6). In contrast to hNSun6, which could only methylate four tRNAs with U73, *Ph*NSun6 could catalyze m^5^C72 formation on eleven *Ph*tRNAs harboring either U73 or G73. We further characterized the tRNA recognition elements for *Ph*NSun6, which were quite different from those of hNSun6. To better understand the recognition mechanism of *Ph*NSun6, we solved the crystal structures of *Ph*NSun6 in the apo form and in complex with SAM, sinefungin (an analog of SAM, SFG), and S-adenosyl-homocysteine (demethylated SAM, SAH). Furthermore, we found that m^5^C72-modified *Ph*tRNAs show slightly higher thermal stability than their unmodified counterparts, but this does not affect the amino acid accepting activity of *Ph*tRNAs. Combined with previous work, we proposed an evolutionary model for eukaryotic and archaeal NSun6 and its tRNA recognition mechanism.

## MATERIALS AND METHODS

### Materials

The m^5^C standard, dithiothreitol (DTT), guanosine monophosphate (GMP), sodium acetate (NaAc), Tris-base, SAH, benzonase, pyrophosphate, and phosphodiesterase I from *Crotalus adamanteus* venom were purchased from Sigma-Aldrich Co. LLC. (St. Louis, MO, USA). Bacterial alkaline phosphatase was obtained from Invitrogen (Shanghai, China). Tris–HCl, tryptone, yeast extract, isopropyl-D-thiogalactoside (IPTG), bovine serum albumin (BSA), MgCl_2_, NaCl, sodium phosphate monobasic, sodium phosphate dibasic, Ethylene diamine tetraacetic acid (EDTA), ATP, CTP, GTP, and UTP were purchased from Sangon Biotech (Shanghai, China). DNA fragment rapid purification and plasmid extraction kits were purchased from Yuanpinghao Biotech (Tianjing, China). KOD-plus mutagenesis kit and KOD-plus-neo Kit were from TOYOBO (Osaka, Japan). Crystallization kits were from Hampton research (Aliso Viejo, CA, USA). T4 DNA ligase, the ribonuclease inhibitor, and all restriction endonucleases were obtained from Thermo Scientific (Waltham, MA, USA). [Methyl-^3^H] SAM (78.0 Ci/mmol) and [^3^H] L-serine were purchased from Perkin Elmer Inc. (Waltham, MA, USA). [^14^C] l-threonine was obtained from Biotrend Chemicals (Destin, FL, USA). SAM was purchased from New England BioLabs (Ipswich, MA, USA). SFG was purchased from Santa Cruz Biotechnology (Santa Cruz, CA, USA). PCR primers were synthesized by BioSune (Shanghai, China). The plasmid pTrc99b was from the Institut de Biologie Moléculaire et Cellulaire du CNRS (Strasbourg, France). The pET28a vector was from Merck–Millipore (Darmstadt, Germany). *Escherichia coli* Rosetta (DE3) cells were purchased from TIANGEN (Beijing, China). Nickel-nitrilotriacetic (Ni-NTA) Superflow resin was purchased from Qiagen, Inc. (Hilden, Germany). The Superdex™ 200 column (10/300 GL; column volume, 23.562 ml) was purchased from GE Healthcare (Fairfield, CT, USA). T7 RNA polymerase was purified from an overproduction strain in our laboratory (38).

### Preparation of tRNA

The *Ph*tRNAs genes were inserted into pTrc99b to construct pTrc99b-T7-*Ph*tRNAs. Mutants of the *Ph*tRNA^Thr^(CGU) were obtained using the KOD-plus mutagenesis kit. All tRNAs were produced using *in vitro* T7 RNA polymerase transcription, as described previously (38). The transcribed tRNAs were separated by urea-denaturing 12% polyacrylamide gel electrophoresis (PAGE), eluted with 0.5 M NaAc (pH 5.2), precipitated with three volumes of ethanol at −20°C and dissolved in 5 mM MgCl_2_. The tRNAs were annealed at 85°C for 10 min and cooled naturally to room temperature for correct folding. The tRNA concentration was determined using UV absorbance at 260 nm, and the molar absorption coefficient was calculated according to the sequence of each tRNA (39).

### Protein expression and purification

The gene encoding PH1991 (*Ph*NSun6) was chemically synthesized by BioSune and inserted into the pET28a vector using NdeI and NotI. *Escherichia coli* Rosetta (DE3) was transformed with the pET28a-*Ph*NSun6 plasmid. Protein overproduction was induced by adding 0.2 mM IPTG into cells. After culturing for 12 h at 18°C, the cells were collected by centrifugation. *Ph*NSun6 was purified by affinity chromatography on Ni-NTA Superflow resin according to the manufacturer's protocol. The wet cells (∼3 g) were suspended in 15 ml of 10 mM imidazole in buffer A [20 mM Tris–HCl (pH 7.5), 500 mM NaCl, 5 mM MgCl_2_, 5 mM DTT and 10% (v/v) glycerol] and sonicated on ice. Then, the sonicated crude extracts were heated at 65°C for 10 min. The crude extracts were centrifuged at 40 000 × *g* for 40 min to remove the debris and insoluble fractions. The supernatant was mixed gently with 0.5 ml Ni-NTA resin for 30 min at 4°C and then washed with 50 ml of 20 mM imidazole in buffer A to remove nonspecific binding proteins. Binding proteins were eluted in 8 ml of 250 mM imidazole in buffer A. The eluted protein was concentrated and purified by gel filtration on a Superdex™ 200 column.

HNSun6 was expressed and purified according to our previous method (33,34). The active *P. horikoshii* SerRS and mouse ThrRS were purified as described previously (40,41). The protein concentrations were determined using UV absorbance at 280 nm, and the molar absorption coefficient was calculated according to the sequence of each protein (42).

### Protein crystallization, structure determination, and refinement

Crystallization was performed at 16°C by the hanging drop vapor diffusion method. For crystallization, *Ph*NSun6 was concentrated to ∼13.3 mg*/*ml. Protein solution (1 μl) was mixed with an equal volume of the reservoir solution, consisting of 0.2 M magnesium acetate tetrahydrate, 0.1 M sodium cacodylate trihydrate pH 6.5 and 20% (w/v) polyethylene glycol 8000. *Ph*NSun6 and 1 mM SAM, or SFG, or SAH, were co-crystallized under the same conditions. The crystals were frozen in liquid nitrogen after transferring for a few seconds in the mother liquor, which contained 15% (v*/*v) glycerol as a cryoprotectant.

All crystal diffraction data sets were collected at the Shanghai Synchrotron Radiation Facility beamlines (SSRF, Shanghai, China) BL-19U1. The diffraction data were processed using the HKL3000 program package (43). Further data analysis was performed with the CCP4 suite (44). The structure of *Ph*NSun6 in the apo form was initially solved by molecular replacement using PHASER (45) with hNSun6 (PDB ID: 5WWQ) structure as starting model. The model was further improved by manual adjustments using COOT (46). The structures of *Ph*NSun6-SAM, *Ph*NSun6-SFG, and *Ph*NSun6-SAH were solved by molecular replacement using PHASER. All models were refined using REFINE program in the PHENIX suite (47). The quality of the final model was evaluated using MOLPROBITY (http://molprobity.biochem.duke.edu/). Figures were drawn using PyMOL (http://www.pymol.org/). A structure-based multiple amino acid sequence alignment of NSun6s from model organisms was generated using ESPript (48).

### tRNA methyl transfer assay

To measure the methyl transfer activity of *Ph*NSun6 and its mutants, 5 μM of tRNAs were used as substrates. The reactions were performed at 65°C under the same conditions in a 25-μl reaction mixture containing 200 μM ^3^H-SAM, 50 mM Tris–HCl (pH 7.5), 200 mM NaCl, 10 mM MgCl_2_, 100 μg/ml BSA, and 5 mM DTT. Reactions were initiated by the addition of 100 nM *Ph*NSun6. Aliquots (5 μl) of the reaction mixtures were removed at time intervals between 2 and 8 min, quenched on Whatman glass-fiber filter discs, and soaked in 5% trichloroacetic acid (TCA). After washing, the amount of radioactive [^3^H]-methyl-tRNA on each disc was measured in a Beckman Las6500 scintillation counting apparatus. The steady-state kinetics were measured under the same conditions with 50–300 nM *Ph*NSun6 and 0.1–40 μM tRNA; the reaction time was 2 or 4 min. For mutants that exhibited extremely low activity, higher concentrations of enzyme and tRNA substrates were used. The data were fitted to Lineweaver-Burk plots, and the *K*_m_, *k*_cat_ and *k*_cat_/*K*_m_ values for each tRNA were calculated from the curve fitting.

To obtain m^5^C72_*Ph*tRNAs, the reactions were performed at 65°C in a mixture comprising 2 μM *Ph*NSun6, 200 μM SAM, 50 mM Tris–HCl (pH 7.5), 200 mM NaCl, 10 mM MgCl_2_, 100 μg*/*ml BSA, and *Ph*tRNAs. The wild-type (WT) *Ph*tRNAs were prepared in the same mixture by adding the *Ph*NSun6 restored buffer. The reaction was performed for 1 h and stopped using phenol/chloroform extraction. The *Ph*tRNAs were precipitated using a 3-fold volume of ethanol.

### Aminoacylation assay

The time course curve for aminoacylation by SerRS for *Ph*tRNA^Ser^ was determined in a 25-μl mixture containing 60 mM Tris–HCl (pH 7.5), 10 mM MgCl_2_, 5 mM DTT, 2.5 mM ATP, 100 μg/ml BSA, 40 μM [^3^H] serine, and 200 nM enzyme, with 5 μM of *Ph*tRNA^Ser^ at 60°C. Aliquots (5 μl) of the reaction mixtures were removed at time intervals between 1 and 4 min, quenched on Whatman glass-fiber filter discs, and soaked in 5% TCA. The time course curve for aminoacylation by ThrRS for *Ph*tRNA^Thr^ was determined at 37°C, as described previously (41).

### Mass spectrometry analysis of m^5^C modification

One microgram of tRNA was hydrolyzed using benzonase, phosphodiesterase I, and bacterial alkaline phosphatase overnight at 37°C in a 20-μl solution including 20 mM Tris–HCl (pH 8.0), 2 mM MgCl_2_, and 20 mM NaCl. The solution was then diluted with H_2_O 100 times and 10 μl was applied to Ultra-Performance Liquid Chromatography-Mass Spectrometry (UPLC-MS). The nucleosides were separated using UPLC on a C18 column (Agilent Zorbax Eclipse Plus C18, 2.1 × 50 mm, 1.8-Micron) and then detected using a triple-quadruple mass spectrometer (Agilent 6400 QQQ) in the positive ion multiple reaction-monitoring (MRM) mode. Mass transition from *m/z* 258 to 126 (m^5^C) was monitored and recorded.

### Melting temperature (*T*_m_) assay of *Ph*tRNAs

Specific tRNAs were dissolved in *T*_m_ buffer [10 mM sodium phosphate monobasic/sodium phosphate dibasic (pH 7.0), 100 μM EDTA and 10 mM NaCl]. The initial absorbance of the tRNA at 260 nm was controlled between 0.2–0.3. A melting temperature curve was measured at 260 nm using a heating rate of 1°C/min from 25 to 95°C via an Agilent Cary 100 spectrophotometer. The *T*_m_ value was determined from the first derivative of the melting curve.

## RESULTS

### PH1991 is the *P. horikoshii* tRNA:m^5^C72 MTase NSun6

PH1991 is the homolog of eukaryotic NSun6 in *P. horikoshii*. To validate whether PH1991 has the tRNA:m^5^C72 MTase catalytic activity, we synthesized the gene that encodes PH1991 and prepared possible tRNA substrates: *Ph*tRNA^Thr^(CGU), *Ph*tRNA^Thr^(GGU), *Ph*tRNA^Thr^(UGU) and *Ph*tRNA^Cys^(GCA), corresponding to the tRNA substrates of hNSun6, using T7 transcription.

**Figure 1.**
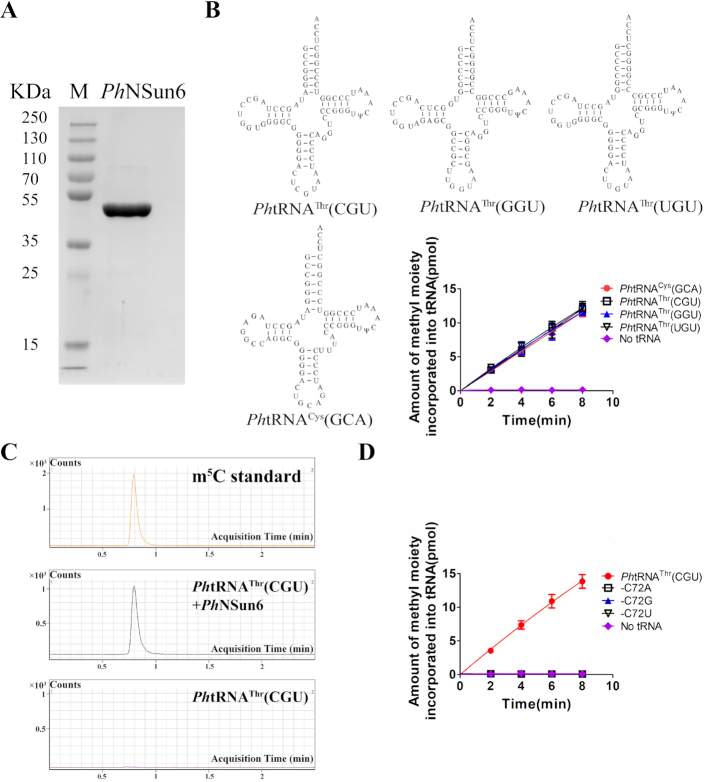
*Ph*NSun6 catalyzes m^5^C72 modification on *Ph*tRNA^Cys^(GCA) and three *Ph*tRNA^Thr^ isoacceptors *in vitro*. (**A**) SDS-PAGE analysis of the purified *Ph*NSun6, as indicated. Standard molecular weights were shown on the left. (**B**) The capacity of *Ph*tRNA^Cys^(GCA) and *Ph*tRNA^Thr^s to be methylated by *Ph*NSun6. (**C**) *Ph*tRNA^Thr^(CGU) incubated with or without *Ph*NSun6 were digested and detected by mass spectrum. (**D**) None of *Ph*tRNA^Thr^(CGU)-C72 mutants could be methylated by *Ph*NSun6. Error bars represent the standard errors of three independent experiments in Figures 1–4.

Subsequently, we purified PH1991 and subjected it to SDS-PAGE analysis (Figure 1A). To assay the MTase activity of PH1991 for *Ph*tRNA^Thr^(CGU), we tested the methyl transfer activity at 37, 55 and 65°C. The highest methyl transfer activity of PH1991 was at 65°C (Supplementary Figure S2). We found that PH1991 was able to catalyze the methylation of three *Ph*tRNA^Thr^ isoacceptors and *Ph*tRNA^Cys^(GCA) at 65°C, similar to hNSun6 at 37°C (Figure 1B). The *Ph*tRNA^Thr^(CGU) methylated by PH1991 were analyzed using UPLC-MS and the modification was confirmed to be m^5^C (Figure 1C), as it was in the methylated *Ph*tRNA^Thr^(GGU), *Ph*tRNA^Thr^(UGU), and *Ph*tRNA^Cys^(GCA) (Supplementary Figure S3). To confirm that C72 was the target site, three mutants, *Ph*tRNA^Thr^(CGU)-C72A, -C72G, and -C72U, were constructed. None of these mutants could be methylated by *Ph*NSun6 (Figure 1D). We measured the steady state kinetic parameters of *Ph*NSun6 in the presence of four *Ph*tRNA substrates (Supplementary Table S1). The *K*_m_ values for the four *Ph*tRNAs were approximately 0.5 μM; however, the *k*_cat_ values showed differences. The *k*_cat_ values of *Ph*NSun6 for three *Ph*tRNA^Thr^ isoacceptors were approximately 4.30 min^−1^, which was higher than that for *Ph*tRNA^Cys^ (3.04 min^−1^). Thus, we identified that PH1991 is the *P. horikoshii* tRNA:m^5^C72 MTase NSun6 and named it *Ph*NSun6.

### *Ph*NSun6 shows a different recognition mechanism for the acceptor stem of tRNAs compared with that of hNSun6

**Figure 2.**
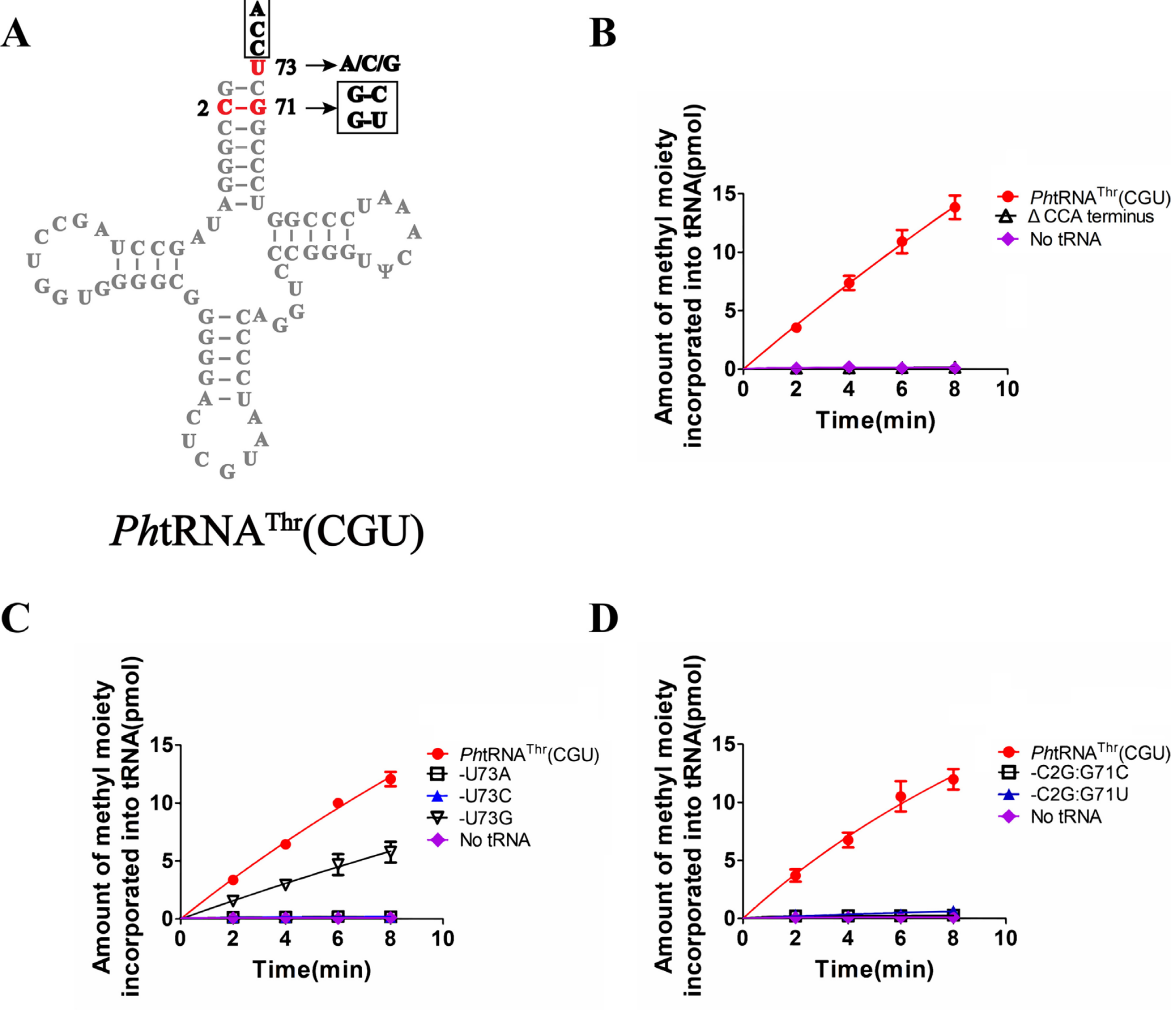
Recognition elements of *Ph*tRNA in the acceptor region by *Ph*NSun6. (**A**) The secondary structure of *Ph*tRNA^Thr^(CGU) summarizing the mutations in the acceptor region. (**B**) The capacity of *Ph*tRNA^Thr^(CGU) mutant lacking CCA terminus to be methylated by *Ph*NSun6. (**C**) The capacity of *Ph*tRNA^Thr^(CGU) with various mutations at position 73 to be methylated by *Ph*NSun6. (**D**) The capacity of *Ph*tRNA^Thr^(CGU) with several mutations in C2:G71 base pair to be methylated by *Ph*NSun6.

Our previous study on hNSun6 showed that the CCA terminus, the target site C72, the discriminator base U73, and base pairs 2:71 and 3:70 in the acceptor stem are the tRNA recognition elements (33). We then determined whether these recognition elements in the acceptor stem were conserved in tRNA recognition by *Ph*NSun6. We assayed the methylation of a *Ph*tRNA^Thr^(CGU) mutant with a deleted CCA terminus and several mutations in U73 and C2:G71 (Figure 2A).

*Ph*NSun6 could not methylate the *Ph*tRNA^Thr^(CGU) mutant lacking the CCA terminus (Figure 2B), implying that the common CCA terminus of tRNA was still essential for the methylation reaction catalyzed by *Ph*NSun6.

To determine whether U73 was necessary for methylation by *Ph*NSun6, we replaced U73 with other the three bases and obtained the *Ph*tRNA^Thr^(CGU)-U73A, -U73C and -U73G mutants (Figure 2A). Remarkably, *Ph*NSun6 could methylate *Ph*tRNA^Thr^(CGU)-U73G, but not *Ph*tRNA^Thr^(CGU)-U73A or -U73C (Figure 2C). Steady-state kinetic assays showed that the *K*_m_ value of *Ph*NSun6 for *Ph*tRNA^Thr^(CGU)-U73G was 0.59 μM, which was slightly higher than that for *Ph*tRNA^Thr^(CGU) (0.43 μM). Whereas the *k*_cat_ value of *Ph*NSun6 for *Ph*tRNA^Thr^(CGU)-U73G was 2.11 min^−1^, which was almost a half that determined for *Ph*tRNA^Thr^(CGU) (4.30 min^−1^) (Supplementary Table S2). These results showed that the efficiency of *Ph*NSun6-mediated methylation of C72 of *Ph*tRNA^Thr^(CGU)-U73G is lower than that for *Ph*tRNA^Thr^(CGU). However, unlike hNSun6, which only recognizes U73, *Ph*NSun6 can recognize both U73 and G73.

According to the tRNA database (31,49), the second base pair in the acceptor stem is C2:G71 or G2:C71 in most *Ph*tRNAs, and in few *Ph*tRNAs is G2:U71; the third base pair exists as C3:G70, G3:C70, A3:U70 and G3:U70. In the four above-mentioned *Ph*tRNAs, the second and third base pairs are C2:G71 and C3:G70, respectively. To determine whether *Ph*NSun6 recognized the second base pair, two mutants, *Ph*tRNA^Thr^(CGU)-C2G:G71C and -C2G:G71U, were constructed and their methylation by *Ph*NSun6 was assessed (Figure 2A). The results showed that *Ph*NSun6 could not methylate these two mutants (Figure 2D; Supplementary Table S2), indicating clearly that C2:G71 is a determinant base pair of tRNA recognition by *Ph*NSun6.

Similarly, we performed C3G:G70C, C3A:G70U and C3G:G70U base pair replacement in *Ph*tRNA^Thr^(CGU). Unexpectedly, *Ph*NSun6 could methylate all these mutants (Supplementary Figure S4 and Table S2), implying that the C3:G70 base pair is not a discriminator for tRNA methylation by *Ph*NSun6.

Collectively, we identified four elements in the acceptor stems of tRNAs that are essential for substrate recognition by *Ph*NSun6: The target site C72, the CCA terminus, U or G73, and the second base pair C2:G71.

### The D-stem is not recognized by *Ph*NSun6

**Figure 3.**
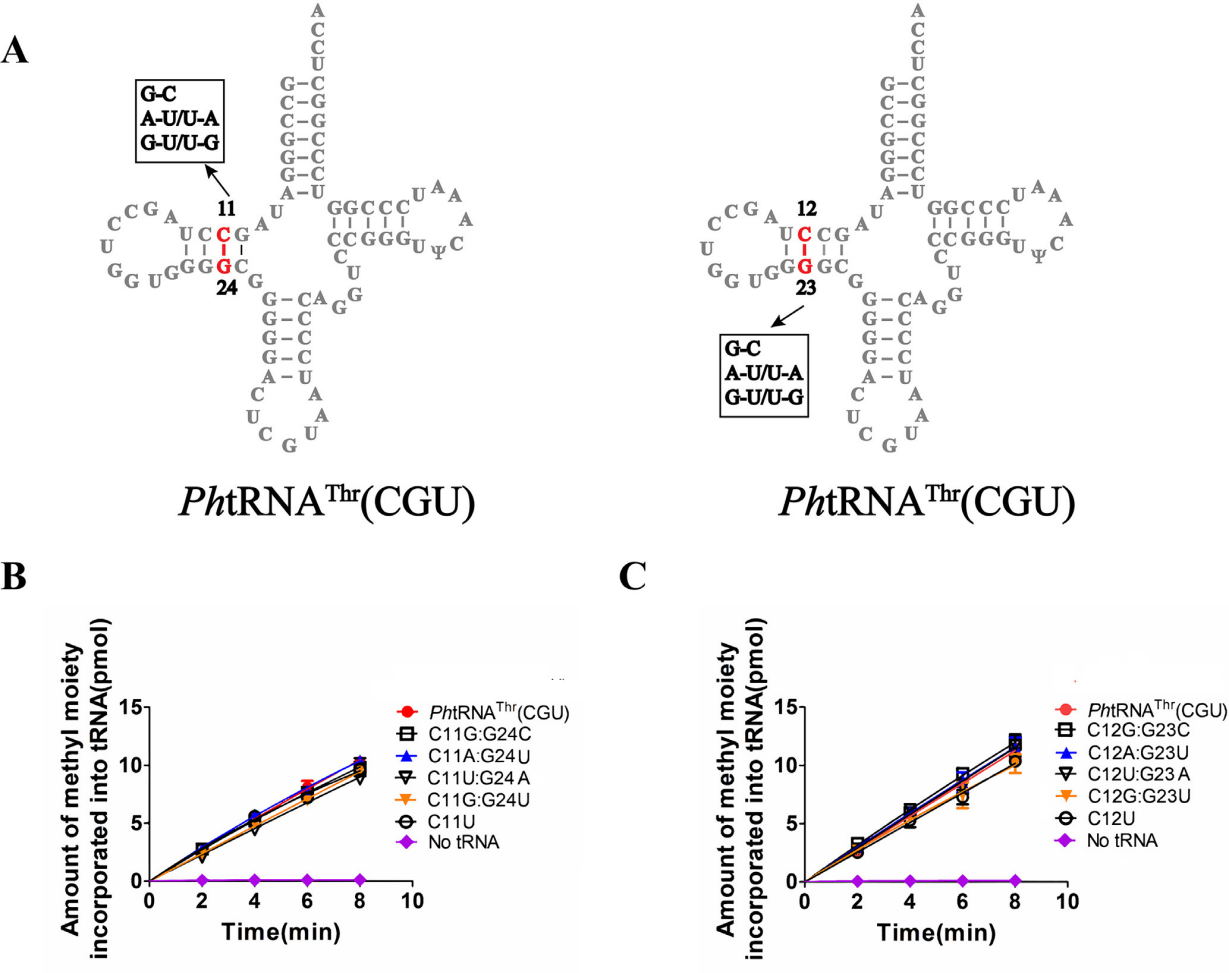
D-stem region is not recognized by *Ph*NSun6. (**A**) The secondary structure of *Ph*tRNA^Thr^(CGU) summarizing the mutations in C11:G24 and C12:G23 base pairs. The capacity of *Ph*tRNA^Thr^(CGU) with various mutations in C11:G24 (**B**) and C12:G23 (**C**) to be methylated by *Ph*NSun6.

Besides elements in the acceptor stem, hNSun6 also interacts with the D-stem of tRNAs (33,34). Base pairs 11:24 and 12:23 of the D-stem are recognized via the eukaryotic NSun6-specific Lys-rich loop (residues ^157^KCKKGAK^163^ in hNSun6, Supplementary Figure S1). However, archaeal NSun6 homologs lack the corresponding eukaryotic NSun6-specific Lys-rich loop, indicating that archaeal NSun6 homologs presumably possess a distinct recognition mechanism in D-stem of tRNA substrates. Therefore, we constructed a series *Ph*tRNA^Thr^(CGU) mutants by replacing C11:G24 or C12:G23 with A:U, U:A, G:C, G:U and U:G, respectively (Figure 3A). Interestingly, these mutants could all be methylated by *Ph*NSun6 (Figure 3B, C), and the *K*_m_ and *k*_cat_ values of *Ph*NSun6 for these mutants were similar to that for *Ph*tRNA^Thr^(CGU) (Supplementary Table S2).

These results suggested that C11:G24 and C12:G23 base pairs in the D-stem are not involved in RNA recognition by *Ph*NSun6.

### *Ph*NSun6 has a wider specificity of tRNA substrates than hNSun6

**Figure 4.**
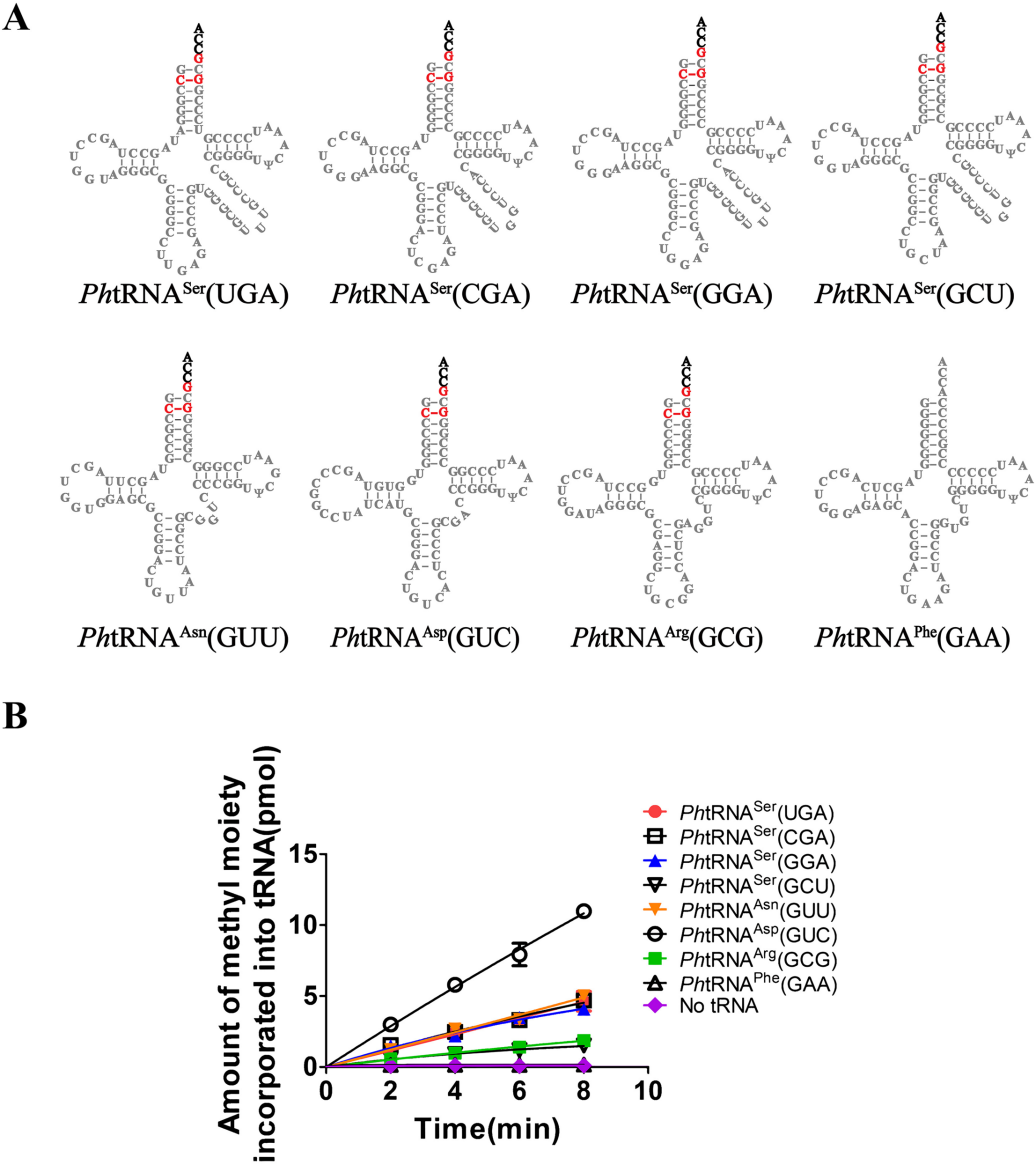
*Ph*NSun6 has wide-range of tRNA substrates. The secondary structures (**A**) and the methylation capacity by *Ph*NSun6 (**B**) of *Ph*tRNA^Ser^(UGA), -(CGA), -(GGA) and -(GCU), *Ph*tRNA^Asn^(GUU), *Ph*tRNA^Asp^(GUC), *Ph*tRNA^Arg^(GCG) and *Ph*tRNA^Phe^(GAA).

Based on the above results, *Ph*NSun6 requires fewer identity elements for tRNA substrates than hNSun6. To examine whether *Ph*NSun6 has other tRNA substrates besides the above four *Ph*tRNAs (three *Ph*tRNA^Thr^ isoacceptors and *Ph*tRNA^Cys^(GCA)), we determined the methylation activity of *Ph*NSun6 for all the *Ph*tRNAs that harbor the recognition elements of *Ph*NSun6. These *Ph*tRNAs are *Ph*tRNA^Ser^(UGA), -(CGA), *-*(GGA) and *-*(GCU), *Ph*tRNA^Asn^(GUU), *Ph*tRNA^Asp^(GUC) and *Ph*tRNA^Arg^(GCG). In addition, *Ph*tRNA^Phe^(GAA) with A73 and G2:C71, which does not match the recognition requirements for *Ph*NSun6, was chosen as a negative control (Figure 4A). Intriguingly, *Ph*NSun6 could catalyze methylation of the above seven tRNAs but not the negative control *Ph*tRNA^Phe^(GAA) (Figure 4B; Supplementary Table S3).

Thus, the results showed that *Ph*NSun6 could efficiently catalyze m^5^C72 formation on eleven *Ph*tRNAs, which suggested that *Ph*NSun6 has a wider range of tRNA substrates than hNSun6.

### Crystal structures of *Ph*NSun6

**Figure 5.**
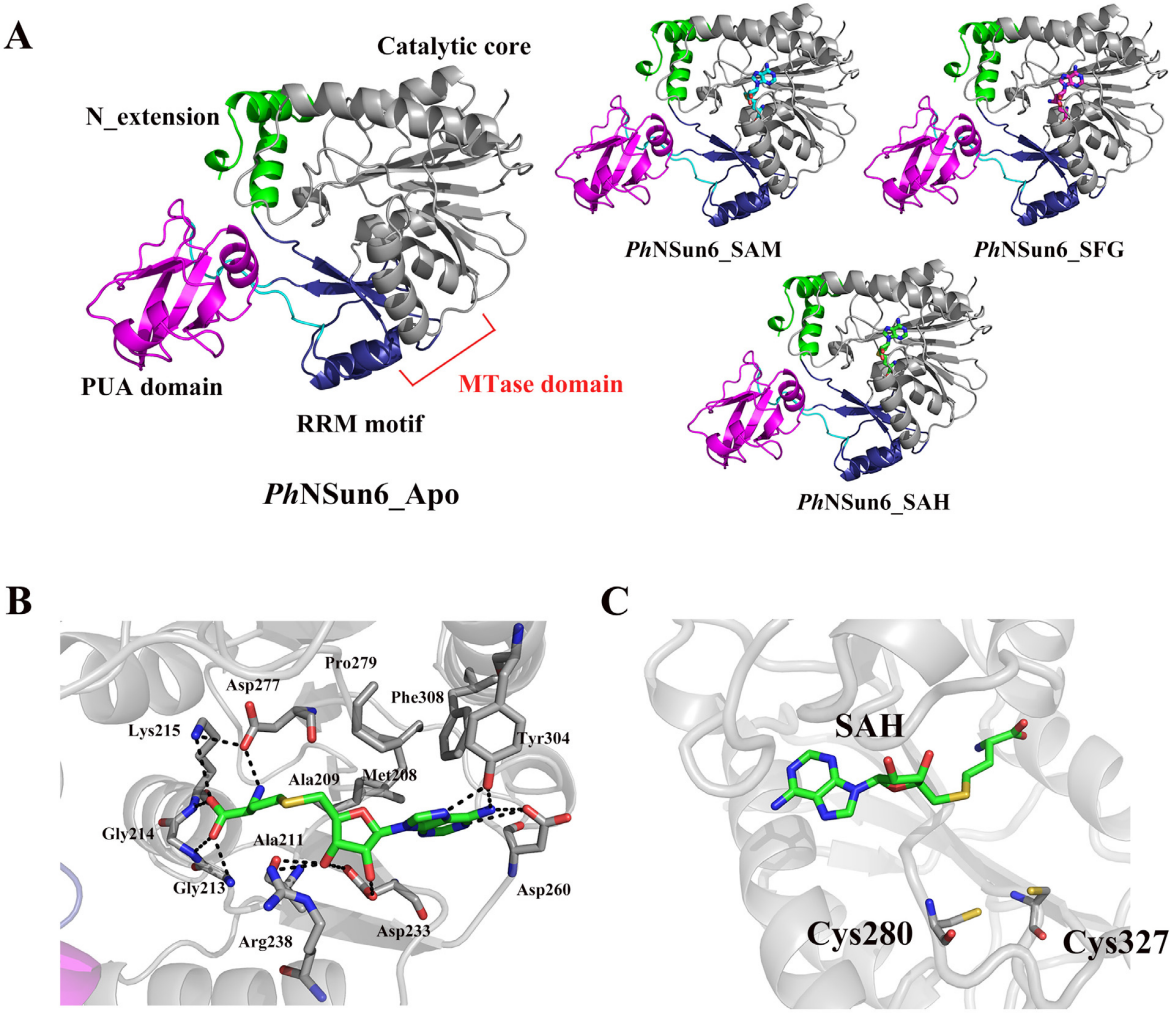
Overall structure of *Ph*NSun6. (**A**) Ribbon diagram showing the overall structure of *Ph*NSun6 in Apo form and in complex with cofactors. The N-terminal extension (green, residues 1–34), linkers (cyan, Linker 1: residues 79–86, and Linker 2: residues 164–173), PUA domain (magenta, residues 86–164) and the MTase domain (residues 34–79 and 173–389) including an RRM motif (dark blue, residues 34–79 and residues 173–185) and a catalytic core (gray, residues 185–389) are highlighted. (**B**) SAH binding details in the *Ph*NSun6-SAH complex. SAH is represented in sticks with green color for carbon atoms, and the hydrogen bonds are indicated as black dotted lines. (**C**) The position of two conserved Cys residues relative to SAH (green, stick representation).

To fully decipher the RNA recognition mechanism of *Ph*NSun6, we obtained the crystal structures of *Ph*NSun6 with and without its cofactors, including apo-*Ph*NSun6, and *Ph*NSun6 complexed with SAM, SFG and SAH, which diffracted to 2.6, 2.5, 2.5 and 2.2 Å, respectively (Table 1). *Ph*NSun6, with 12 α-helices and 19 β-strands, is divided into two distinct domains with different sizes: the MTase domain, including an RRM motif (residues 34–79 and residues 173–185) and a Rossmann-fold catalytic core (residues 185–389), and the PUA domain (residues 86–164). The PUA domain is inserted into the MTase domain via two linkers: Residues 79–86 and residues 164–173 (Figure 5A). A non-conserved extension motif (residues 1–34) was observed at the N-terminus.

**Table 1.** Data collection and refinement statistics

Measurement	*Ph*NSun6-Apo	*Ph*NSun6-SAH	*Ph*NSun6-SAM	*Ph*NSun6-SFG
**Data collection**				
Space group	*P*1	*P*1	*P*1	*P*1
Cell dimensions				
*a, b, c* (Å)	52.19, 52.60, 83.968	52.98, 52.17, 83.804	52.11, 52.98, 83.801	52.47, 52.94, 84.18
*α*, *β*, *γ* (°)	81.78, 81.78, 67.93	80.63, 80.58, 67.90	80.80, 80.75, 67.84	81.49, 81.38, 67.45
Resolution (Å)^a^	50–2.60 (2.64–2.60)	50–2.18 (2.22–2.18)	50–2.50 (2.54–2.50)	50–2.50 (2.54–2.50)
*R*_sym_	11.5 (39.2)	13.3 (42.1)	10.9 (33.4)	11.2 (32.7)
*I* / σ*I*	14.2 (3.0)	12.4 (2.4)	16.2 (3.7)	17.3 (3.4)
Completeness (%)	91.4 (67.0)	93.4 (73.8)	91.9 (70.7)	92.2 (67.1)
Redundancy	6.7 (5.4)	6.5 (4.6)	6.6 (4.9)	6.0 (4.4)
**Refinement**				
Resolution (Å)	48.1–2.60	41.1–2.18	48.8–2.50	41.4–2.50
No. reflections (work/free)	22105/1064	39519/1997	25054/1259	25193/1285
*R*_work_/*R*_free_	0.177/0.236	0.189/0.236	0.179/0.236	0.182/0.229
No. atoms				
Protein	6156 (2 chains)	6156 (2 chains)	6156 (2 chains)	6156 (2 chains)
Ligand		52 (2xSAH)	54 (2xSAM)	54 (2xSFG)
*B*-factors				
Protein	37.9	31.6	36.8	36.8
Ligand		30.4	39.3	41.1
Bond lengths (Å)	0.005	0.004	0.005	0.005
Bond angles (°)	0.889	0.901	0.925	0.925

^a^Values in parentheses are for the highest-resolution shell.

Cofactors bind at the Rossmann-fold catalytic core of *Ph*NSun6. When comparing the structures of *Ph*NSun6 in the apo form and in the form bound with cofactors, the overall structures do not show distinct changes, except that helix α9 moves a slightly toward the cofactors (Supplementary Figure S5). Residues Met208, Ala209, Pro279, Phe308, Asp260, Asp233, Arg238, Ala211, Asp277, Lys215, Tyr304, Gly213 and Gly214 form the binding pocket for the cofactor SAH, as shown in Figure 5B. These residues are mostly conserved or semi-conserved in eukaryotic and archaeal NSun6s, except for the archaeal-specific residue Tyr304, which recognizes N6 and N7 of the adenine ring of SAH. In the *Ph*NSun6-SAM and *Ph*NSun6-SFG complexes, similar binding models of the cofactors to that of SAH were observed, but are not discussed here.

Although *Ph*NSun6 contains a different RNA recognition motif compared with that in hNSun6, the main structure of *Ph*NSun6 adopts a similar topology to hNSun6 and the two active Cys residues of *Ph*NSun6 are conserved. The two conserved Cys residues point from opposite site sides of the cofactor, which is the same as that in hNSun6 (Figure 5C).

**Figure 6.**
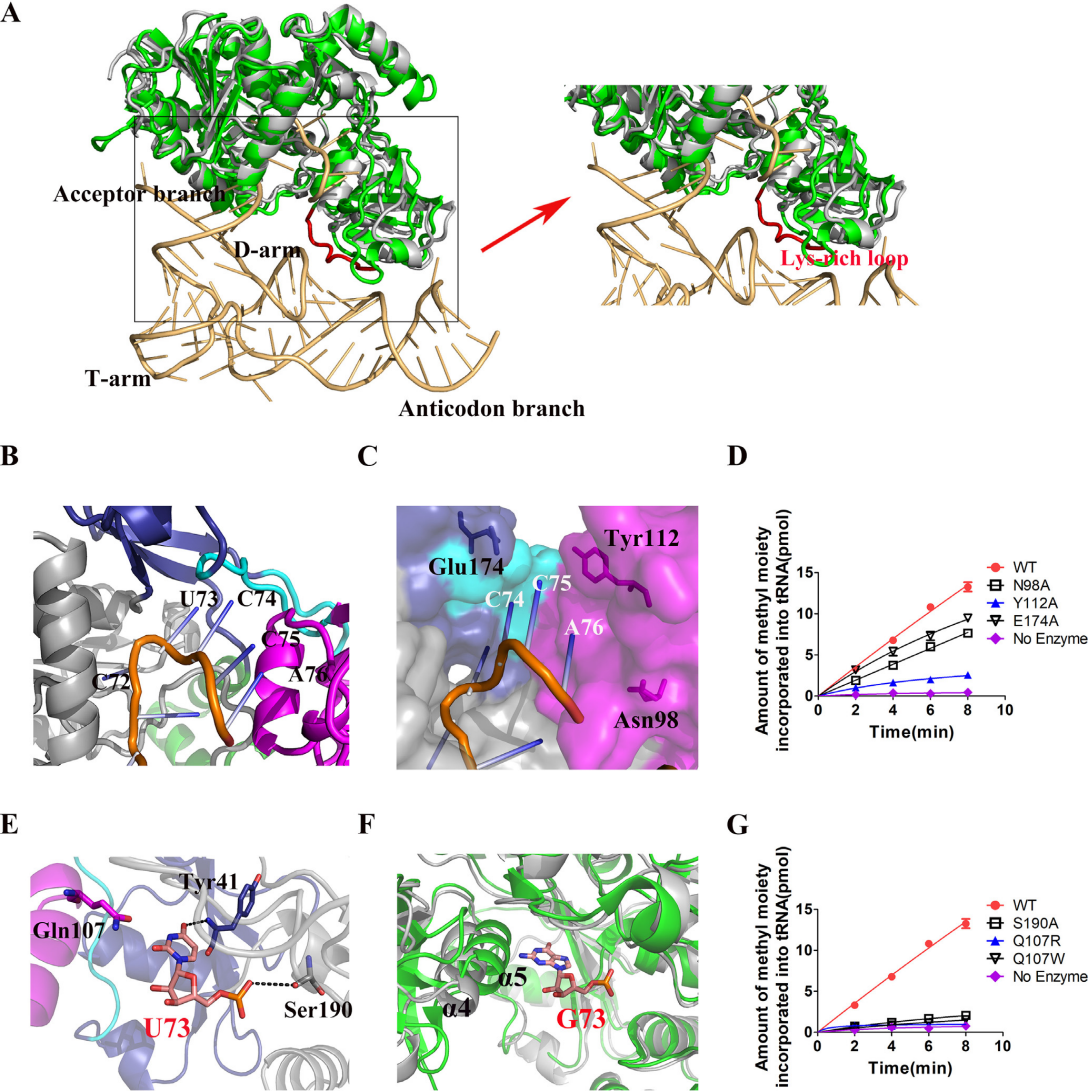
Binding model of *Ph*NSun6 with tRNA. (**A**) Structural superposition of *Ph*NSun6/SAH (gray) and hNSun6/tRNA/SFG (green) and the tRNA backbone is shown in light orange. HNSun6-specific Lys-rich loop is marked as red. The CCA terminus of tRNA binds to the linkers and PUA domain in the *Ph*NSun6/tRNA superposition model presented in cartoon (**B**) and surface representations (**C**). (**E**) The proposed recognition residues of U73 (salmon, stick representation) by *Ph*NSun6. (**F**) The proposed recognition details of G73 (salmon, stick representation) by *Ph*NSun6 (gray), comparing to that by hNSun6 (green). α4 helix of *Ph*NSun6 and α5 helix of hNSun6 are highlighted. (**D**) and (**G**) depict the methylation activities of *Ph*NSun6 and its variants.

Some structural differences were observed between *Ph*NSun6 and hNSun6. The first difference is that hNSun6 contains two unique insertions (hNSun6 specific insertion 1 and 2) in the surface of MTase catalytic core (Supplementary Figure S6). Second, for the PUA domain, most important difference concerns the change in the orientations of *Ph*NSun6 helix α4 and hNSun6 α5 (Figure 6F). Third, *Ph*NSun6 lacks the eukaryotic specific Lys-rich loop (Figure 6A). Taken together, the structural differences between *Ph*NSun6 and hNSun6 might contribute to the distinct tRNA substrate recognition mechanisms.

### Binding model of *Ph*NSun6 with tRNA

We were not able to solve the structure of the *Ph*NSun6 in complex with its tRNA substrate. Thus, we generated a tRNA binding model of *Ph*NSun6 by superimposing the structure of *Ph*NSun6/SAH onto that of the hNSun6/SFG/tRNA complex (PDB ID: 5WWR) (34). In this model, the PUA domain of *Ph*NSun6 has no interactions with the D-stem region of tRNA, because of the lack of the eukaryotic NSun6-specific Lys-rich loop (Figure 6A), which is consistent with D-stem of tRNA not being recognized by *Ph*NSun6.

The CCA terminus of tRNA is essential for *Ph*NSun6 recognition. According to the *Ph*NSun6/tRNA model, all the three nucleotides of the CCA terminus interact extensively with residues from the PUA domain and linkers (Figure 6B, C). The binding mode between the CCA terminus and *Ph*NSun6 is very similar to that observed from the hNSun6/tRNA complex. However, as mentioned above, the PUA domain, especially those residues on the protein surface, are quite divergent between *Ph*NSun6 and hNSun6. To test this superposition model, we mutated putative CCA terminus-interacting residues (Asn98, Glu174 and Tyr112) of *Ph*NSun6 to Ala. All the three mutants showed reduced methyltransferase activities compared with WT protein. The *K*_m_ values of *Ph*NSun6-N98A and -E174A for *Ph*tRNA^Thr^(CGU) were ∼2-fold higher than that of *Ph*NSun6, while their *k*_cat_ values remain similar (Figure 6D; Supplementary Table S4); the *K*_m_ value of *Ph*NSun6-Y112A for *Ph*tRNA^Thr^(CGU) was 5.3-fold higher than that of *Ph*NSun6 while its *k*_cat_ value decreased (Figure 6D; Supplementary Table S4). These results suggest that all the three residues of *Ph*NSun6 contribute to tRNA binding. Thus, we conjectured that the PUA domain has retained the CCA binding capability during evolution.

The hypothetical binding pocket of *Ph*NSun6 for the base at the 73^rd^ nucleotide of the *Ph*tRNA substrate comprises Ser190, Gln107, and Tyr41 (Figure 6E), which are absolutely conserved in archaeal NSun6 homologs. Intriguingly, the archaeal-specific hypothetical binding pocket for N73 seems propitious to accommodate either U or G, mainly thanks to the orientation of helix α4, which enlarges the size of the pocket in *Ph*NSun6. In contrast, the corresponding helix α5 in hNSun6 limits the binding pocket to U73 only (Figure 6F). In the *Ph*NSun6/tRNA model, the O4 of U73 or O6 of G73 could form a hydrogen bond with the main chain amide group of Tyr41. However, when U73/G73 is substituted by C73 or A73, the N4 of C73 or N6 of A73 could not form this hydrogen bond, thus tRNAs with C73 or A73 will be excluded by *Ph*NSun6. The archaeal-specific residues in the hypothetical binding pocket for N73 might explain why *Ph*NSun6 could accommodate U or G, but not C or A, at site 73 of tRNA substrates. To test our hypothesis, we made mutations on Ser190 and Gln107 of *Ph*NSun6. The OH group from the side chain of Ser190 forms a hydrogen bond to the phosphate group of N73. When Ser190 was mutated to Ala, *Ph*NSun6-S190A had little methylation activity compared to *Ph*NSun6 (Figure 6G). On the other hand, in the hypothetical binding model, side chain of Gln107 is close to the uracil ring of U73. To modify the size and the charge of the binding pocket for the base ring of N73, Gln107 was replaced by Trp with large side chain or by Arg with positive charge respectively. Both *Ph*NSun6-Q107W and -Q107R mutants lost methylation activity completely (Figure 6G). These results suggest that residues in the proposed N73 binding pocket are important for methylation of *Ph*NSun6.

### The m^5^C72 modification of tRNA does not affect the aminoacylation activity of aminoacyl-tRNA synthetase (aaRS)

Considering that the m^5^C72 modification is close to the CCA terminus, we investigated whether the m^5^C72 modification affected the amino acid accepting activity of tRNA by the corresponding aaRS. We tested seven (*Ph*tRNA^Ser^(CGA), *-*(GGA), *-*(UGA) and *-*(GCU), *Ph*tRNA^Thr^(CGU), -(GGU) and *-*(UGU)) of the eleven *Ph*tRNAs. The aminoacylation activity of aaRS for the m^5^C72-modified *Ph*tRNAs showed no obvious difference compared that for their unmodified counterparts (Supplementary Figure S7). These results suggest that the m^5^C72 modification does not affect the amino acid accepting activity of the tested *Ph*tRNAs.

### The m^5^C72 modification slightly increases the thermal stability of *Ph*tRNAs

Archaea usually exist in extreme environments, such as hot springs and salt lakes. *P. horikoshii* is a hyperthermophilic anaerobic archaeon, growing optimally at 98°C (50). Critically, according to statistical analyses, archaeal NSun6 homologs only exist in hyperthermophilic archaea.

In hyperthermophilic organisms, tRNAs have to tolerate high temperature, resulting in the development of certain strategies to protect tRNAs from degradation and denaturation, and to increase the stability of tRNA tertiary structure, such as high GC-content and tRNA modifications (16,18,51). Notably, loss of some tRNA modifications in hyperthermophilic tRNAs affects their thermal stability. For example, in *Thermus thermophilus*, lack of the m^7^G46 modification causes decreased *T*_m_ values of class I tRNAs and degradation of tRNA^Phe^ and tRNA^Ile^, resulting in reduced protein synthesis at high temperatures (52).

To determine whether there is a correlation between the m^5^C72 modification and the thermal stability of tRNAs, we measured the *T*_m_ of the eleven m^5^C72-modified *Ph*tRNAs and their unmodified counterparts. The data were shown in Table 2. Except for *Ph*tRNA^Arg^(GCG), the *T*_m_ values for the other ten *Ph*tRNAs with the m^5^C72 modifications all increased compared with those for their unmodified counterparts. However, the delta *T*_m_ values are between ∼1 and 2°C in most cases. Only *Ph*tRNA^Asp^(GUC) showed a 3°C difference between methylated and unmethylated tRNAs. The results suggested that m^5^C72 contributes thermal stability towards tRNA, although the effect from this single modification is small.

**Table 2.** Melting temperatures (*T_m_*) of *Ph*tRNAs or m^5^C72-modified *Ph*tRNAs

tRNAs	*T_m_*(°C) *Ph*tRNAs	*T_m_*(°C) m^5^C72-modified *Ph*tRNAs	Δ*T_m_*(°C)
*Ph*tRNA^Thr^(CGU)	66.3±0.4	67.3±0.4	1.0
(GGU)	61.3±0.4	62.3±0.4	1.0
(UGU)	69.3±0.5	71.3±0.5	2.0
*Ph*tRNA^Cys^(GCA)	61.2±0.5	62.8±0.5	1.6
*Ph*tRNA^Ser^(UGA)	62.7±0.4	64.7±0.5	2.0
(CGA)	65.8±0.4	66.8±0.4	1.0
(GGA)	67.5±0.4	69.5±0.6	2.0
(GCU)	65.2±0.5	66.8±0.5	1.6
*Ph*tRNA^Asn^(GUU)	71.6±0.5	73.0±0.6	1.4
*Ph*tRNA^Asp^(GUC)	73.2±0.9	76.2±0.5	3.0
*Ph*tRNA^Arg^(GCG)	68.0±0.1	68.7±0.5	0.7

Each of melting temperature was calculated from the average of three independent measurements and standard deviation values were also shown.

### Archaeal or eukaryotic NSun6 distinguish their cognate tRNA substrates

Based on this work and our previous work on eukaryotic NSun6 (33,34), a remarkable aspect of NSun6 is the difference in substrate specificity between archaeal and eukaryotic homologs (23,33). This raises an interesting question of whether archaeal or eukaryotic NSun6 could discriminate each other's tRNA substrates.

**Figure 7.**
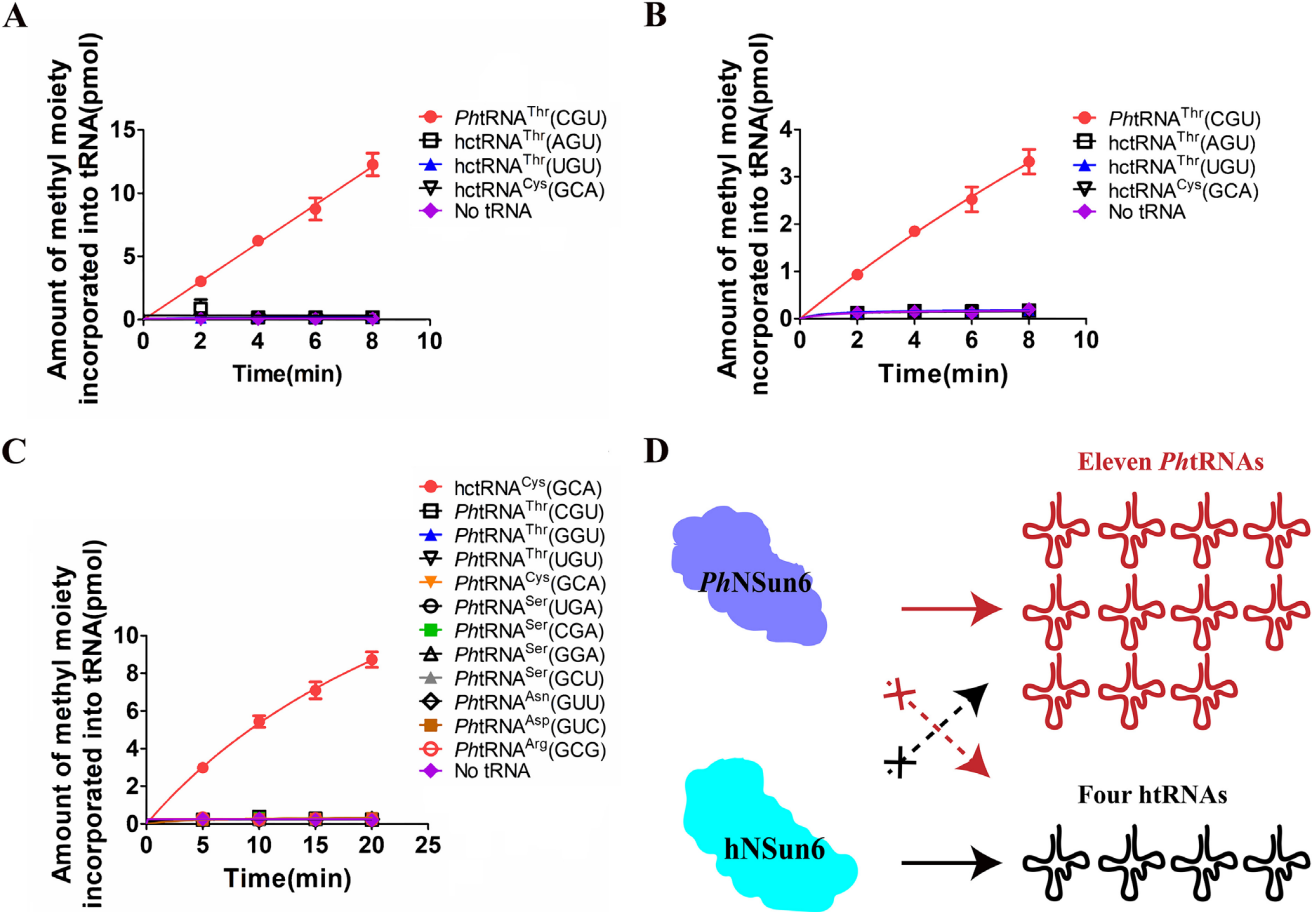
*Ph*NSun6 or hNSun6 discriminates the cognate tRNA substrates. The capacity of *Ph*tRNA^Thr^(CGU) and three hctRNAs to be methylated by *Ph*NSun6 at 65°C (**A**) or 55°C (**B**). (**C**) The capacity of hctRNA^Cys^(GCA) and eleven *Ph*tRNAs to be methylated by hNSun6. Error bars represent the standard errors of three independent experiments. (**D**) Schema showing that *Ph*NSun6 or hNSun6 could only methylate the cognate tRNA substrates.

First, we verified whether the tRNA substrates of hNSun6 could be methylated by *Ph*NSun6. Considering the stability of hctRNAs, we tested the methyl transferase activity of *Ph*NSun6 to hctRNA^Thr^(AGU), hctRNA^Thr^(UGU), and hctRNA^Cys^(GCA) at 65 or 55°C. The results showed that the tRNA substrates of hNSun6 could not be methylated by *Ph*NSun6 at 65°C (Figure 7A) or 55°C (Figure 7B). Considering that C2:G71 is the determinant element for *Ph*NSun6 recognition, we speculated that the tRNA substrates of hNSun6 were not distinguished by *Ph*NSun6 because these native tRNAs harbor a G2:C71 base pair.

We also tested the methylation of the eleven *Ph*NSun6 tRNA substrates by hNSun6. The results showed that none of the eleven tRNA substrates of *Ph*NSun6 could be catalyzed by hNSun6 (Figure 7C). For hNSun6 recognition, tRNAs with U73 are strictly recognized. The seven *Ph*tRNAs with G73: *Ph*tRNA^Ser^(UGA), *-*(CGA), *-*(GGA) and -(GCU), *Ph*tRNA^Asn^(GUU), *Ph*tRNA^Asp^(GUC) and *Ph*tRNA^Arg^(GCG) should not be methylated by hNSun6. Our previous study showed that the tRNA^Cys^(GCA)-G2C:C71G mutant is not a substrate of hNSun6 (33). Therefore, the four U73 containing *Ph*tRNAs: *Ph*tRNA^Thr^(CGU), *-*(GGU) and -(UGU), and *Ph*tRNA^Cys^(GCA) with the C2:G71 base pair could not be methylated by hNSun6 for this reason. These results rationalized the identified tRNA recognition elements for *Ph*NSun6 and hNSun6 (33).

In conclusion, *Ph*NSun6 or hNSun6 could only methylate its own cognate tRNA substrates, and the two enzymes could not recognize each other's cognate tRNA substrates (Figure 7D).

## DISCUSSION

### tRNA recognition mechanism by archaeal NSun6s

In this study, we identified that PH1991 is the *P. horikoshii* tRNA:m^5^C72 MTase NSun6, *Ph*NSun6. The tRNA substrates recognized by *Ph*NSun6 were based on (i) the 3′ CCA terminus, (ii) the target site C72, (iii) U73 or G73, and (iv) the discriminatory base pair C2:G71 at the acceptor stem. The CCA terminus and the target site C72 are common recognition elements for *Ph*NSun6 and hNSun6. However, they exist in almost all tRNAs, which are not enough to distinguish substrate tRNAs of NSun6. U73/G73 and C2:G71 function as discriminatory elements to identify *Ph*NSun6 substrate tRNAs from nonsubstrate tRNAs.

In the superimposition model of *Ph*NSun6/tRNA, the binding pocket of site 73 is capable of accommodating both U and G, which was in line with our methylation experiment in which eleven *Ph*tRNAs with U73 or G73 could be substrates of *Ph*NSun6. In the model, the base moiety of G71 was not in contact with *Ph*NSun6. However, based on our results, C2:G71 functions as a discriminatory base pair that can select substrate tRNAs from other *Ph*tRNAs. The exact recognition mechanism of *Ph*NSun6 for C2:G71 requires further mechanistic and structural studies.

Based on the *Ph*NSun6/tRNA superimposition model, *Ph*NSun6 does not interact with the D-stem region of the tRNA, because of the lack of the eukaryotic NSun6-specific Lys-rich loop, which is consistent with biochemical data on the effect of the D-stem on activity of *Ph*NSun6. Thus, our biochemical and crystallographic data suggest that *Ph*NSun6 ignores the sequential specificity of the D-stem of *Ph*tRNA substrates. Considering that archaeal NSun6 homologs share high identity with each other, we speculated that the tRNA substrate specificity of *Ph*NSun6 is conserved in other archaeal NSun6 homologs.

### A proposed evolutionary model for NSun6

**Figure 8.**
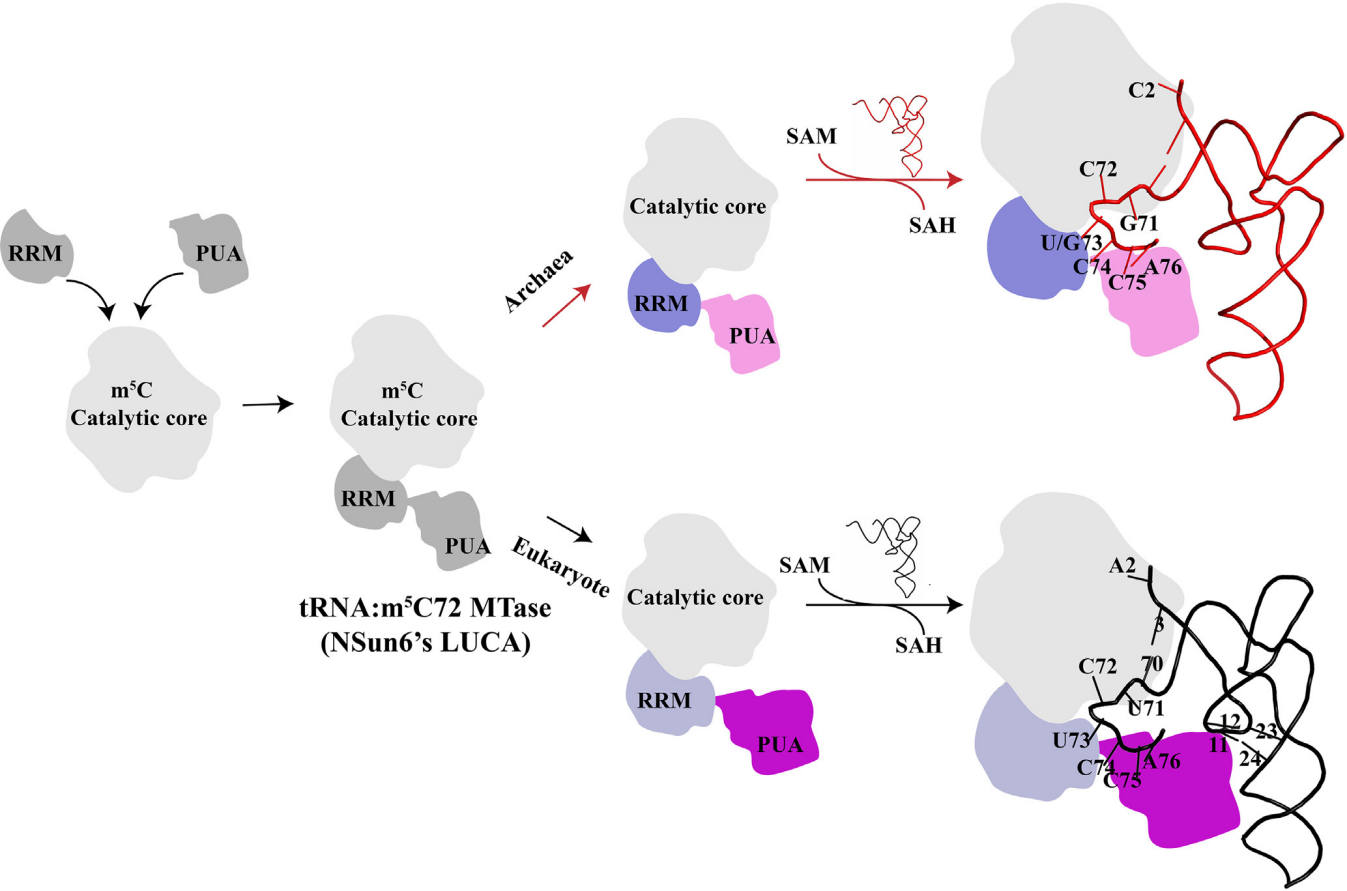
A proposed evolutionary model for NSun6. Originally, ancient m^5^C catalytic core recruited and inserted RRM motif and PUA domain, resulting in NSun6’s LUCA. Later in evolution, NSun6 divided into two branches: archaeal NSun6 and eukaryotic NSun6. Due to the divergent RRM motif and PUA domain during evolution, archaeal and eukaryotic NSun6 exhibit different discrimination mechanisms for the cognate tRNA substrates. For RNA substrates recognition, eukaryotic NSun6 recognizes the acceptor stem and D-stem region, while archaeal NSun6 only interacts with the acceptor stem region. The tRNA elements that are recognized by archaeal or eukaryotic NSun6 are summarized here and highlighted in sticks.

Based on the results of this study, together with previous findings on hNSun6, we proposed an evolutionary model for NSun6 (Figure 8). Originally, the RRM and PUA domain were recruited and inserted into an ancient m^5^C catalytic core to bind RNA substrates, resulting in the formation of NSun6’s last universal common ancestor (LUCA). Specifically, for the recognition by NSun6’s LUCA of tRNA substrates, the PUA domain recognized the common CCA terminus; the RRM recognized site 73; and the m^5^C catalytic core recognized C72 and base pairs near site 72. Later in evolution, NSun6 divided into two branches: Archaeal NSun6 and eukaryotic NSun6. The divergence of the RRM and PUA domain during evolution, which increased the precision and accuracy of RNA substrate discrimination, archaeal and eukaryotic NSun6 exhibit different discrimination mechanisms for its cognate tRNA substrates. For RNA substrate discrimination, eukaryotic NSun6 recognizes the acceptor stem and D-stem, while archaeal NSun6 only interacts with the acceptor stem. The evolutionary difference between archaeal and eukaryotic NSun6 has led to diverse cognate tRNA recognition mechanisms.

### Biological functions of m^5^C72 modification

The presence of m^5^C modifications in RNA has been observed across the three domains of life. However, the tRNA:m^5^C modification is present only in archaea and eukaryotes (31,32,53). The m^5^C modification could increase hydrophobicity and reinforce stacking interactions, probably leading to tRNA stabilization (54). In *Saccharomyces cerevisiae*, the m^5^C40 modification of tRNA^Phe^ stabilizes the tRNA anticodon stem (55); lack of m^5^C48 and m^5^C49, together with m^7^G46, cause the rapid decay of certain tRNAs (56).

In archaea, NSun6s are present only in hyperthermophilic species (57). In terms of tRNA canonical functions, m^5^C72 modification does not affect the amino acid accepting activity of tRNAs, but slightly increases the thermal stability of *Ph*tRNAs. Acceptor stem of tRNA is rarely modified, and m^5^C72 is one of the few modifications in this region. It remains possible that m^5^C72 has other biological functions *in vivo*, which awaits further studies on both archaeal and eukaryotic NSun6s.

## DATA AVAILABILITY

Protein Data Bank: atomic coordinates and structure factors for apo *Ph*NSun6 have been deposited with accession code 5ZVD; for the *Ph*NSun6-SAM, *Ph*NSun6-SFG, and *Ph*NSun6-SAH complex under accession code 5ZVG, 5ZVH and 5ZVE, respectively.

## Supplementary Material

Supplementary Data
