# Supplementary material for: Archaeal NSUN6 catalyzes m5C72 modification on a wide-range of specific tRNAs
Source: Nucleic Acids Res. 2018 Dec 12;47(4):2041–55. doi: 10.1093/nar/gky1236 (PMC6393295; doi:10.1093/nar/gky1236)
Supplement: Supplementary Data [file gky1236_supplemental_files.pdf]

SUPPLEMENTARY DATA

Table S1. Kinetic parameters of *Ph*NSun6 for *Ph*tRNA^{Cys} and *Ph*tRNA^{Thr}s in the methyl transfer reaction

tRNAs	K_m (µM)	k_{cat} (min^{-1})	k_{cat}/K_m (min^{-1} µM^{-1})
*Ph*tRNA^{Cys}(GCA)	0.38±0.02	3.04±0.12	8.02
*Ph*tRNA^{Thr}(CGU)	0.43±0.03	4.30±0.15	10.00
(GGU)	0.63±0.02	4.34±0.43	6.60
(UGU)	0.59±0.03	4.43±0.28	7.52

All parameters represent the average of three independent experiments with standard deviations indicated in table 1-4, and ND stands for not detectable.

Table S2. Kinetic parameters of *Ph*NSun6 for *Ph*tRNA^{Thr}(CGU) with mutations in the methyl transfer reaction

tRNA domain	*Ph*tRNA^{Thr}(CGU)	K_m (µM)	k_{cat} (min^{-1})	k_{cat}/K_m (min^{-1} µM^{-1})
	WT	0.43±0.03	4.30±0.15	10.00
Acceptor stem	-C72A	ND	ND	ND
	-C72G	ND	ND	ND
	-C72U	ND	ND	ND
	-deleted CCA	ND	ND	ND
	-U73A	ND	ND	ND
	-U73C	ND	ND	ND
	-U73G	0.59±0.05	2.11±0.19	3.58
	-C2G:G71C	ND	ND	ND
	-C2G:G71U	ND	ND	ND
	-C3G:G70C	0.91±0.11	2.85±0.12	3.13
	-C3A:G70U	1.07±0.10	7.26±0.37	6.79
	-C3G:G70U	0.63±0.01	4.37±0.28	6.94
D-stem	-C11G:G24C	0.56±0.01	4.35±0.23	7.77
	-C11A:G24U	0.39±0.01	4.17±0.03	10.69
	-C11U:G24A	0.67±0.05	4.31±0.21	6.43
	-C11G:G24U	0.57±0.01	4.11±0.19	7.21
	-C11U	0.65±0.04	5.44±0.13	8.37
	-C12G:G23C	0.50±0.06	4.24±0.38	8.48
	-C12A:G23U	0.58±0.04	4.30±0.90	7.35
	-C12U:G23A	0.55±0.02	4.39±0.63	8.01
	-C12G:G23U	0.51±0.02	5.01±0.19	9.89
	-C12U	0.55±0.01	4.14±0.16	7.53

Table S3. Kinetic parameters of *Ph*NSun6 for more *Ph*tRNAs in the methyl transfer reaction

tRNAs	K_m (μM)	k_{cat} (min^{-1})	k_{cat}/K_m (min^{-1} $μM^{-1}$)
*Ph*tRNA^{Ser}(UGA)	0.83±0.05	2.23±0.25	2.68
(CGA)	0.76±0.02	1.50±0.05	1.97
(GGA)	0.64±0.05	1.19±0.06	1.86
(GCU)	0.56±0.03	0.37±0.04	0.66
*Ph*tRNA^{Asn}(GUU)	0.70±0.07	1.76±0.06	2.51
*Ph*tRNA^{Asp}(GUC)	0.30±0.00	3.71±0.20	12.67
*Ph*tRNA^{Arg}(GCG)	0.52±0.06	0.67±0.02	1.29
*Ph*tRNA^{Phe}(GAA)	ND	ND	ND

Table S4. Kinetic parameters of *Ph*NSun6's mutants for *Ph*tRNA^{Thr}(CGU) in the methyl transfer reaction

***Ph*NSun6**	K_m (μM)	k_{cat} (min^{-1})	k_{cat}/K_m (min^{-1} $μM^{-1}$)
WT	0.43±0.03	4.30±0.15	10.00
N98A	0.93±0.13	4.11±0.32	4.42
E174A	0.95±0.19	4.04±0.56	4.25
Y112A	2.27±0.36	0.85±0.02	0.37

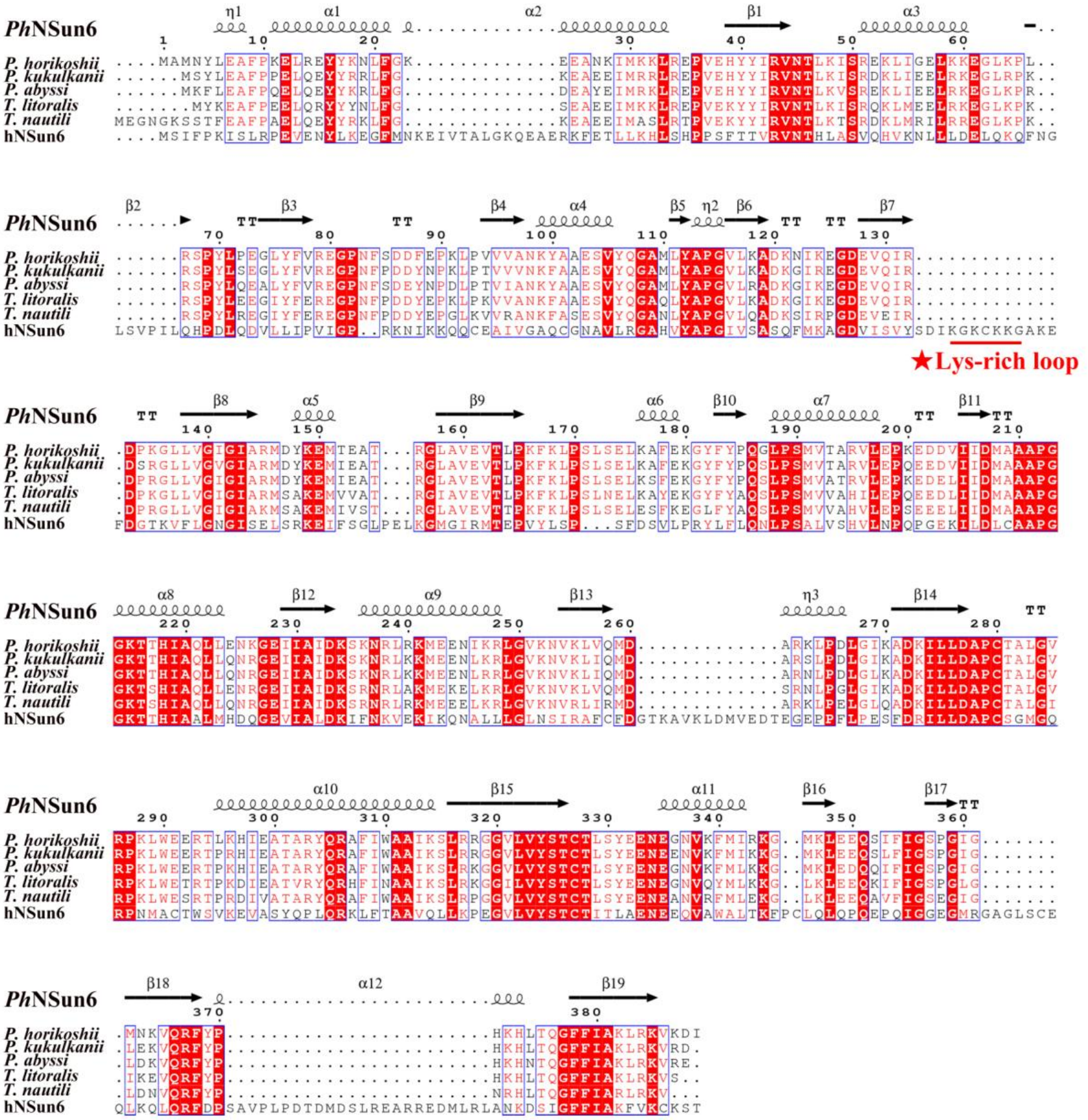

Figure S1. Sequence alignment of archaeal NSun6s.

Structure-based multiple amino acid sequence alignment of archaeal NSun6 homologs from model organisms. *P. horikoshii*, *Pyrococcus horikoshii* OT3; *P. kukulkanii*, *Pyrococcus kukulkanii* NCB100; *P. abyssi*, *Pyrococcus abyssi* GE5; *T. litoralis*, *Thermococcus litoralis* NS-C; *T. nautili*, *Thermococcus nautili* 30-1; hNSun6, human NSun6. The secondary structure elements of *Ph*NSun6 are labeled above the alignment. The specific Lys-rich loop (^{157}KCKKGAK^{163}) in hNSun6 is marked below the alignment.

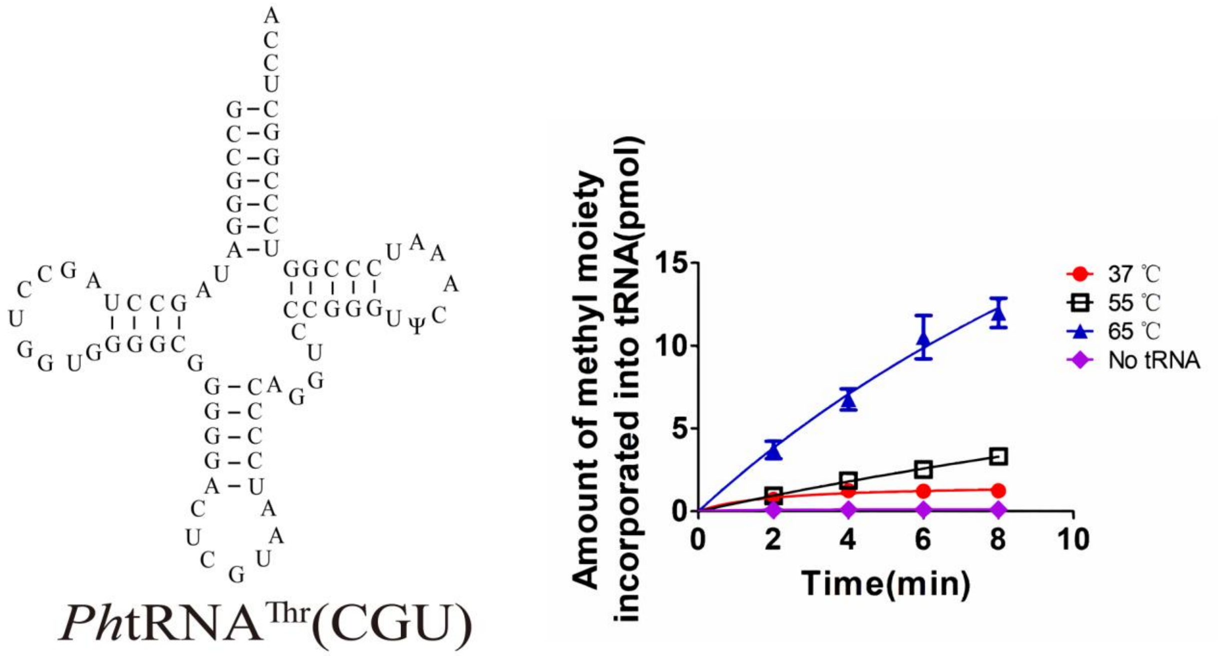

Figure S2. The methyl transfer activity of *Ph*NSun6 to *Ph*tRNA^{Thr}(CGU) incubated at different temperature.

Amount of ^{3}H(Met) incorporation into *Ph*tRNA^{Thr}(CGU) incubated at different temperature. Error bars represent the standard errors of three independent experiments.

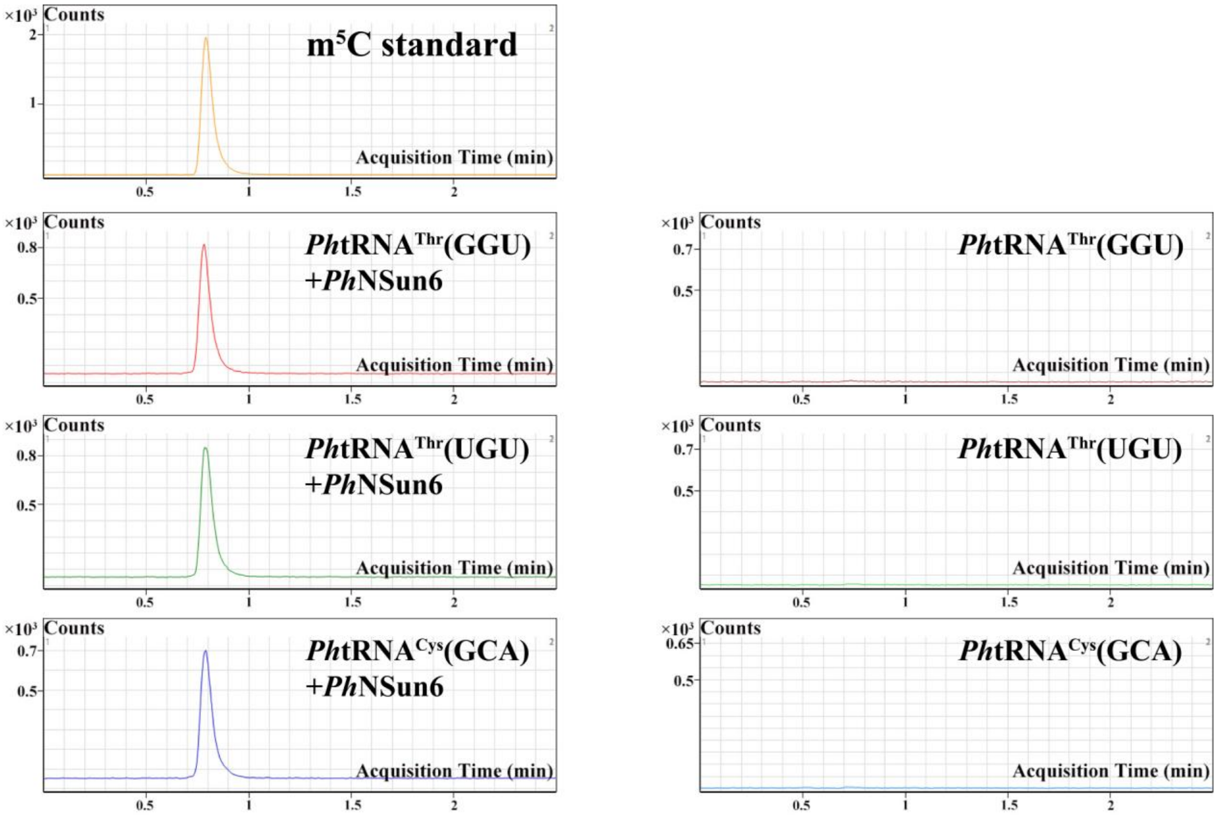

Figure S3. ***Ph*****NSun6 catalyzes m^{5}C72 formation on** ***Ph*****tRNA^{Thr}(GGU),** ***Ph*****tRNA^{Thr}(UGU) and** ***Ph*****tRNA^{Cys}(GCA).**

*Ph*tRNA^{Thr}(GGU), *Ph*tRNA^{Thr}(UGU) and *Ph*tRNA^{Cys}(GCA) incubated with or without *Ph*NSun6 were digested and detected by mass spectrum.

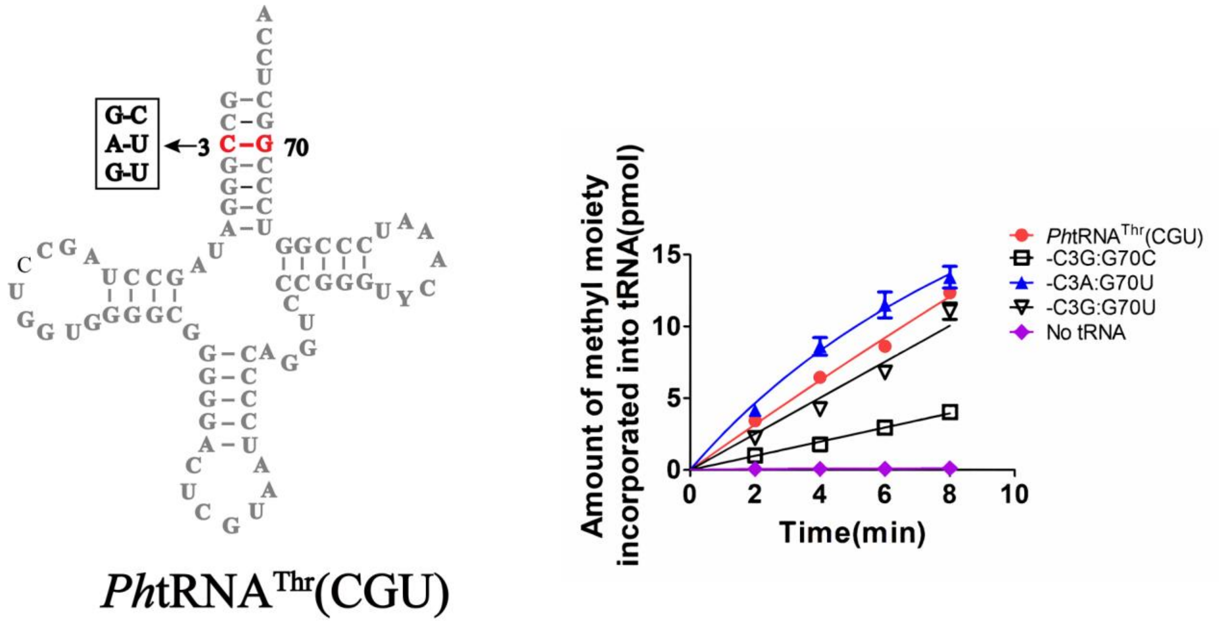

Figure S4. The capacity of *Ph*tRNA^{Thr}(CGU) with various mutations in C3:G70 base pair to be methylated by *Ph*NSun6.

The capacity of *Ph*tRNA^{Thr}(CGU) with several mutations in C3:G70 base pair to be methylated by *Ph*NSun6. Error bars represent the standard errors of three independent experiments.

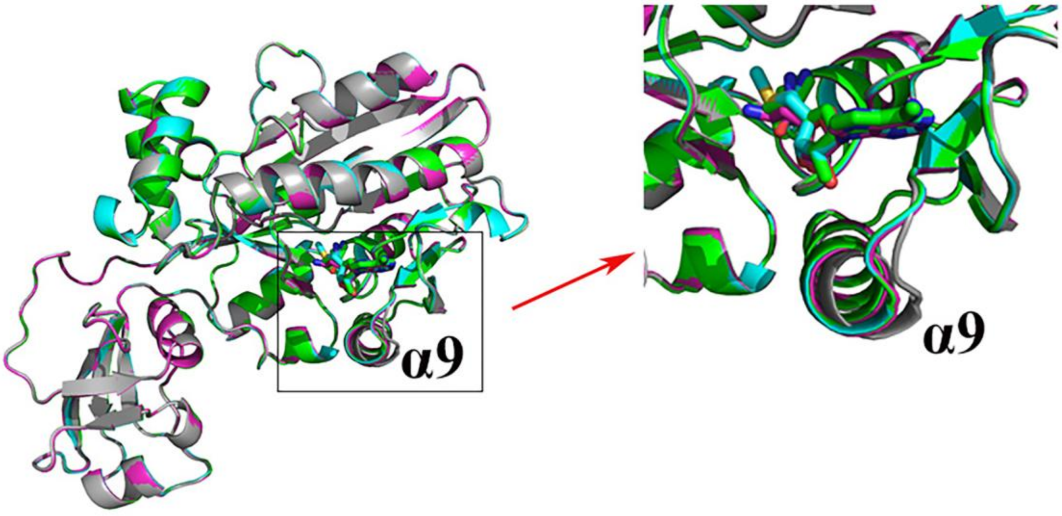

Figure S5. The structural differences between *Ph*NSun6 in apo form and in the form of binding with cofactors.

Superposition of the *Ph*NSun6-apo (gray), *Ph*NSun6-SAH (green), *Ph*NSun6-SAM (cyan), and *Ph*NSun6-SFG (magenta) crystal structures reveal differences in the orientation of helix α9 of chain B.

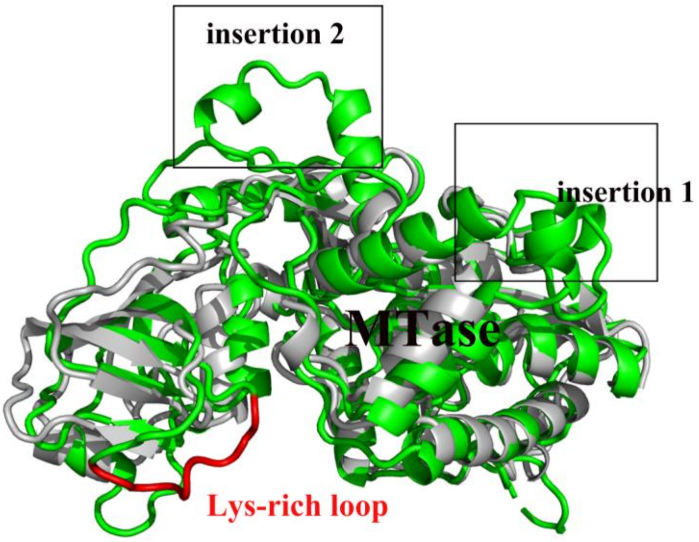

Figure S6. Structural superposition of *Ph*NSun6-SAH (gray) and hNSun6-apo (green).

Two hNSun6 specific insertions in the surface of MTase catalytic core are highlighted as box annotation and hNSun6-specific Lys-rich loop is marked as red.

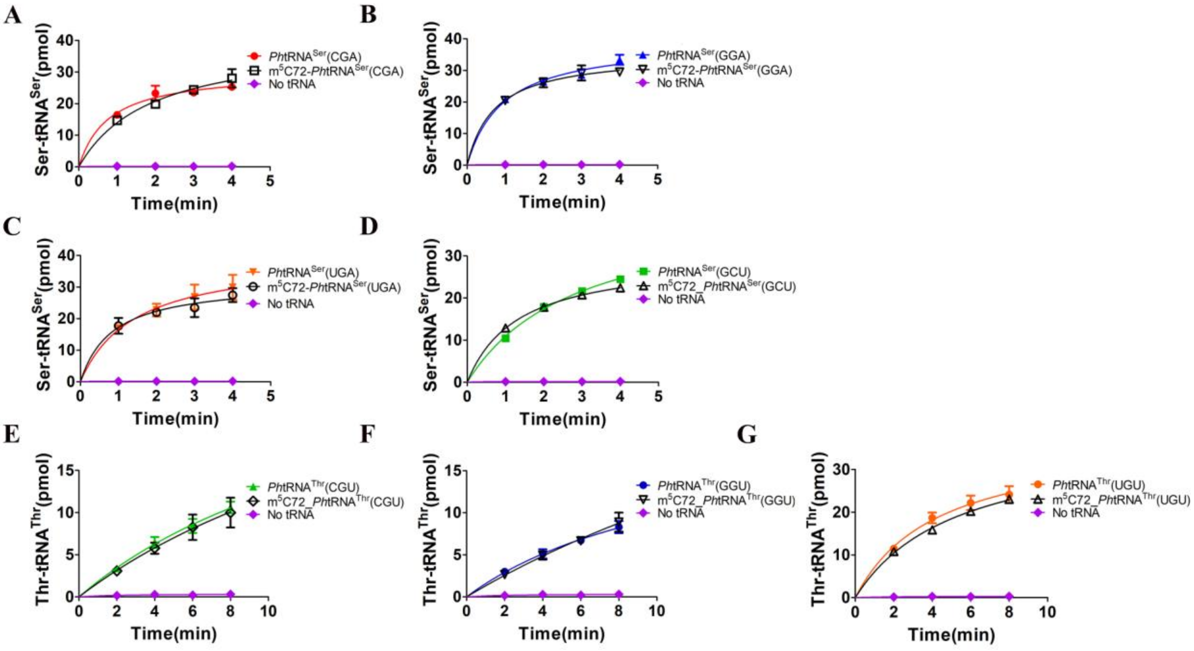

Figure S7. The aminoacylation activities of aaRS for tRNA or m^{5}C72-modified tRNA.

(A, B, C and D) Time course curves of aminoacylation of *Ph*tRNA^{Ser}s and m^{5}C72-modified *Ph*tRNA^{Ser}s by SerRS. (E, F and G) Time course curves of aminoacylation of *Ph*tRNA^{Thr}s and m^{5}C72-modified *Ph*tRNA^{Thr}s by ThrRS. Error bars represent the standard errors of three independent experiments.
